# Supplementary material for: Combined Plasma and Urinary Metabolomics Uncover Metabolic Perturbations Associated with Severe Respiratory Syncytial Viral Infection and Future Development of Asthma in Infant Patients
Source: Metabolites. 2022 Feb 14;12(2):178. doi: 10.3390/metabo12020178 (PMC8875115; doi:10.3390/metabo12020178)
Supplement: Supplementary file 1 [file metabolites-12-00178-s001.zip › metabolites-1532822-suppl-revised.pdf]

**Supplementary Table S1. Patient demographics of RSV Study 2018–2019 (N=30).**

| | (n) | % | mean | median | st.dev. |
|---|---|---|---|---|---|
| **Weight (kg)** | | | | | |
| Controls | 10 | | 6.81 | 7.32 | 1.76 |
| RSV | 20 | | 4.61 | 4.25* | 1.65 |
| **Ethnicity** | | | | | |
| Controls | 10 | | | | |
| Caucasian/European | 9 | 90.0 | | | |
| Black or Hispanic | 1 | 10.0 | | | |
| RSV | 20 | | | | |
| Caucasian/European | 17 | 85.0 | | | |
| Black or Hispanic | 3 | 15.0 | | | |
| **Premature** (< 36 weeks) | 3 | | | | |
| Controls | 0 | 0.0 | | | |
| RSV | 3 | 15.0 | | | |

*Mann-Whitney Rank test control vs. RSV at time of enrollment p-value ≤ 0.005.

**Supplementary Table S2. Diagnosis of RSV positive patients (N=20).**

| | (n) | mean | median | st.dev. |
|---|---|---|---|---|
| **Diagnosis*** | | | | |
| Acute bronchiolitis | 20 | | | |
| RSV | 20 | | | |
| Acute respiratory failure | 20 | | | |
| Pneumonia[a] | 15 | | | |
| Viral | 10 | | | |
| Bacterial | 11 | | | |
| Anemia, unspecified | 2 | | | |
| Atelectasis | 2 | | | |
| Other[b] | 12 | | | |
| **Severity of Illness Scores** | | | | |
| PELOD Comprehensive | 19 | 10.70 | 11.00 | 3.28 |
| Day 0[c] | 19 | 8.63 | 11.00 | 4.21 |
| Severe | 15 | 8.87 | 11.00 | 4.09 |
| Moderate | 4 | 7.75 | 10.00 | 5.19 |
| Day 3[d] | 19 | 6.78 | 11.00 | 5.45 |
| Severe | 15 | 7.93 | 11.00 | 5.05 |
| Moderate | 4 | 2.75 | 0.00 | 5.50 |
| PIM2 | 20 | -5.13 | -5.03 | 0.92 |
| Severe | 15 | -5.004 | -4.9 | 0.94 |
| Moderate | 5 | -5.51 | -5.92 | 0.83 |
| PRISM III | 20 | 2.15 | 1.5 | 1.95 |
| Severe | 15 | 2.13 | 1.0 | 2.03 |
| Moderate | 5 | 2.2 | 2.0 | 1.92 |

*Patients may have more than one diagnosis code listed. [a]Includes: Pneumonia due to other specified bacteria; Pneumonia, unspecified organism. [b]Includes: Failure to thrive (child). [c]14/19 patients scored 1 for PELOD Pulmonary score. [d]11/19 patients scored a 1 for PELOD Pulmonary score.

**Supplementary Table S3. PLS coefficients of plasma metabolites in RSV model.**

| Metabolite | Coefficient vs RSV severity |
|---|---|
| Xanthosine | 0.0536 |
| Phosphoserine | 0.0527 |
| Nacetylneuraminate | 0.0501 |
| NAD | 0.0413 |
| CoA | 0.0411 |
| GAP.DHAP | 0.0384 |
| 2.hydroxyglutarate | 0.0372 |
| Serine | 0.0323 |
| Allantoate_and_Carbamoyl_aspartate | 0.0318 |
| GDP | 0.0312 |
| Proline | 0.0306 |
| FBP | 0.0293 |
| Glycine | 0.0291 |
| GMP | 0.0290 |
| dCMP | 0.0267 |
| Uracil | 0.0243 |
| Glutathione_reduced | 0.0229 |
| UDP.D.glucuronate | 0.0217 |
| GDP.fucose | 0.0199 |
| Glutamine | 0.0198 |
| GDP.mannose | 0.0189 |
| ADP | 0.0177 |
| Threonine | 0.0165 |
| Hexose.phosphate | 0.0158 |
| Glutathione_disulfide_oxidized | 0.0150 |
| Acetyl.CoA | 0.0150 |
| AMP | 0.0139 |
| ADP.ribose | 0.0131 |
| UMP | 0.0120 |
| UDP.D.glucose | 0.0102 |
| Citrulline | 0.0100 |
| Ribose.5.phosphate | 0.0097 |
| Valine | 0.0080 |
| xylulose.5.phosphate | 0.0077 |
| Histidine | 0.0075 |
| Asparagine | 0.0069 |
| Alanine | 0.0068 |
| Lysine | 0.0036 |
| Glutamic.acid | 0.0024 |
| Methionine | 0.0018 |

| | |
|---|---|
| IMP | 0.0016 |
| Leucine | 0.0006 |
| Bisphosphoglycerate | -0.0013 |
| Ribulose.5.phosphate | -0.0022 |
| Phenylalanine | -0.0029 |
| Ornithine | -0.0030 |
| Aspartic.acid | -0.0051 |
| Isoleucine | -0.0053 |
| ATP | -0.0056 |
| Succinate | -0.0082 |
| Phosphoenolpyruvate | -0.0084 |
| Phosphogluconic_acid | -0.0096 |
| Arginine | -0.0100 |
| Tryptophan | -0.0104 |
| CMP | -0.0116 |
| Malate | -0.0117 |
| Fumarate | -0.0250 |
| UDP.N.acetylglucosamine | -0.0277 |
| Tyrosine | -0.0301 |
| Citrate.isocitrate | -0.0326 |
| CMP.N.acetylneuraminate | -0.0392 |
| Ketoglutarate | -0.0408 |
| Glycerol_3.phosphate | -0.0500 |
| N.acetylglucosamine.1.phosphate | -0.0727 |

**Supplementary Table S4. PLS coefficients of urinary metabolites in RSV model.**

| Metabolite | Coefficient vs RSV severity |
|---|---|
| AMP | 0.0528 |
| UMP | 0.0503 |
| xylulose.5.phosphate | 0.0432 |
| IMP | 0.0426 |
| UDP.N.acetylglucosamine | 0.0298 |
| CMP | 0.0294 |
| Ornithine | 0.0290 |
| GDP.fucose | 0.0264 |
| UDP.D.glucose | 0.0253 |
| Glutathione_reduced | 0.0245 |
| GDP.mannose | 0.0229 |
| Hexose.phosphate_1 | 0.0197 |
| Citrulline | 0.0193 |
| Nacetylneuraminate | 0.0192 |
| UTP | 0.0180 |
| GMP | 0.0177 |
| ADP.ribose | 0.0169 |
| Glycine | 0.0163 |
| NAD | 0.0156 |
| Lysine | 0.0146 |
| Proline | 0.0140 |
| Asparagine | 0.0133 |
| Tyrosine | 0.0102 |
| Hexose.phosphate_2 | 0.0088 |
| NADH | 0.0084 |
| Arginine | 0.0078 |
| Acetyl.CoA | 0.0075 |
| NADPH | 0.0068 |
| Threonine | 0.0057 |
| Leucine | 0.0042 |
| CTP | 0.0036 |
| dCMP | -0.0005 |
| Aspartic.acid | -0.0006 |
| NADP | -0.0016 |
| Serine | -0.0018 |
| Glutamic.acid | -0.0023 |
| Glutathione_disulfide_oxidized | -0.0024 |
| GAP.DHAP | -0.0026 |
| Glutamine | -0.0041 |
| FAD | -0.0053 |

| | |
|---|---|
| Valine | -0.0054 |
| Alanine | -0.0054 |
| UDP.D.glucuronate | -0.0055 |
| Xanthosine | -0.0058 |
| UDP | -0.0060 |
| Histidine | -0.0060 |
| Methionine | -0.0080 |
| Phenylalanine | -0.0083 |
| Uracil | -0.0110 |
| Malate | -0.0122 |
| FBP | -0.0124 |
| Isoleucine | -0.0175 |
| Hexose.phosphate_3 | -0.0197 |
| Ribose.5.phosphate | -0.0197 |
| Glycerol_3.phosphate | -0.0209 |
| Tryptophan | -0.0216 |
| ADP | -0.0225 |
| 2.3.phosphoglycerate | -0.0247 |
| 2.hydroxyglutarate | -0.0247 |
| Flavin_mononucleotide | -0.0249 |
| Phosphoserine | -0.0282 |
| Succinate | -0.0328 |
| Ribulose.5.phosphate | -0.0341 |
| CMP.N.acetylneuraminate | -0.0380 |
| Bisphosphoglycerate | -0.0382 |
| N.acetylglucosamine.1.phosphate | -0.0412 |
| Phosphogluconic_acid | -0.0420 |
| CDP | -0.0551 |
| GDP | -0.0602 |
| Citrate.Isocitrate | -0.0675 |

**Supplementary Table S5. PCA loadings in the covariate model.**

| | PC1 | PC2 |
|---|---|---|
| Weight | -0.350 | 0.232 |
| PL.protein | -0.337 | 0.082 |
| Age_wks | -0.272 | 0.049 |
| Birth_weight | -0.165 | 0.385 |
| Race | -0.062 | -0.229 |
| Diet | 0.008 | -0.213 |
| UR.protein | 0.105 | 0.162 |
| Gender | 0.113 | -0.207 |
| Lymph | 0.120 | 0.034 |
| Timepoint | 0.130 | -0.034 |
| Eosinophils | 0.133 | -0.286 |
| Prematurity | 0.164 | -0.371 |
| WBC | 0.164 | 0.282 |
| Neu | 0.167 | 0.335 |
| ENT_problems | 0.198 | 0.401 |
| Classified_As_asthmatic | 0.239 | 0.189 |
| Pathology | 0.336 | 0.038 |
| Length_of_stay | 0.372 | 0.097 |
| Length_of_vent | 0.396 | 0.010 |

**Supplementary Table S6. PLS coefficients of metabolites in asthma prediction model.**

| Metabolite | Coefficient vs asthma |
|---|---|
| UR.Lysine | 0.0664 |
| PL.Uracil | 0.0634 |
| UR.Ornithine | 0.0590 |
| UR.UMP | 0.0581 |
| UR.IMP | 0.0579 |
| UR.GDP.mannose | 0.0557 |
| PL.Asparagine | 0.0511 |
| UR.CTP | 0.0477 |
| PL.Serine | 0.0470 |
| PL.Arginine | 0.0453 |
| PL.Citrulline | 0.0432 |
| UR.Asparagine | 0.0421 |
| PL.Threonine | 0.0414 |
| PL.UDP.D.glucuronate | 0.0406 |
| PL.Methionine | 0.0397 |
| UR.AMP | 0.0347 |
| UR.GDP.fucose | 0.0335 |
| UR.NADPH | 0.0323 |
| PL.Lysine | 0.0321 |
| UR.Hexose.phosphate_2 | 0.0303 |
| PL.CMP | 0.0300 |
| PL.Glutamine | 0.0291 |
| PL.Glycine | 0.0284 |
| PL.Isoleucine | 0.0284 |
| PL.Fumarate | 0.0281 |
| PL.GDP.mannose | 0.0275 |
| PL.Nacetylneuraminate | 0.0268 |
| UR.Malate | 0.0250 |
| UR.Glycine | 0.0248 |
| PL.Phosphogluconic_acid | 0.0233 |
| UR.Acetyl.CoA | 0.0233 |
| UR.Proline | 0.0230 |
| UR.Succinate | 0.0228 |
| PL.GDP.fucose | 0.0225 |
| UR.ADP.ribose | 0.0206 |
| PL.UDP.D.glucose | 0.0186 |
| UR.Arginine | 0.0166 |
| PL.Phosphoserine | 0.0165 |
| PL.Tyrosine | 0.0152 |
| PL.Hexose.phosphate | 0.0145 |

| | |
|---|---|
| UR.UDP.N.acetylglucosamine | 0.0138 |
| PL.Aspartic.acid | 0.0133 |
| PL.Allantoate_and_Carbamoyl_aspartate | 0.0131 |
| PL.Proline | 0.0129 |
| PL.Glutamic.acid | 0.0129 |
| PL.FBP | 0.0109 |
| UR.GAP.DHAP | 0.0105 |
| UR.Threonine | 0.0100 |
| PL.Bisphosphoglycerate | 0.0099 |
| PL.CMP.N.acetylneuraminate | 0.0098 |
| PL.Alanine | 0.0094 |
| PL.Glutathione_disulfide_oxidized | 0.0092 |
| PL.Phosphoenolpyruvate | 0.0088 |
| PL.Tryptophan | 0.0087 |
| PL.ATP | 0.0084 |
| UR.Phosphogluconic_acid | 0.0082 |
| UR.UDP.D.glucose | 0.0081 |
| PL.Acetyl.CoA | 0.0067 |
| PL.Ribose.5.phosphate | 0.0063 |
| PL.UDP.N.acetylglucosamine | 0.0062 |
| UR.Phosphoserine | 0.0055 |
| UR.UTP | 0.0049 |
| PL.Ribulose.5.phosphate | 0.0049 |
| PL.Leucine | 0.0045 |
| UR.Flavin_mononucleotide | 0.0042 |
| UR.Ribulose.5.phosphate | 0.0037 |
| PL.xylulose.5.phosphate | 0.0029 |
| UR.NAD | 0.0026 |
| UR.Glutathione_disulfide_oxidized | 0.0025 |
| PL.UMP | 0.0020 |
| PL.NAD | 0.0016 |
| UR.Uracil | 0.0015 |
| UR.Ribose.5.phosphate | 0.0010 |
| PL.AMP | 0.0009 |
| UR.Histidine | 0.0007 |
| UR.Leucine | 0.0006 |
| PL.GDP | 0.0001 |
| PL.ADP.ribose | -0.0012 |
| PL.Ornithine | -0.0015 |
| PL.dCMP | -0.0024 |
| UR.Glutamine | -0.0029 |
| UR.Serine | -0.0030 |
| PL.Glutathione_reduced | -0.0030 |

| | |
|---|---|
| UR.Tyrosine | -0.0040 |
| UR.FAD | -0.0040 |
| PL.ADP | -0.0047 |
| PL.Xanthosine | -0.0048 |
| UR.Aspartic.acid | -0.0057 |
| UR.UDP | -0.0058 |
| UR.Alanine | -0.0075 |
| UR.Citrate.Isocitrate | -0.0083 |
| UR.NADP | -0.0103 |
| UR.CMP | -0.0107 |
| UR.xylulose.5.phosphate | -0.0118 |
| UR.GMP | -0.0119 |
| PL.Ketoglutarate | -0.0122 |
| UR.Phenylalanine | -0.0124 |
| UR.Glutathione_reduced | -0.0128 |
| UR.Isoleucine | -0.0139 |
| PL.Valine | -0.0152 |
| PL.GMP | -0.0153 |
| UR.CDP | -0.0153 |
| UR.Glycerol_3.phosphate | -0.0165 |
| UR.2.hydroxyglutarate | -0.0166 |
| UR.N.acetylglucosamine.1.phosphate | -0.0174 |
| PL.2.hydroxyglutarate | -0.0174 |
| UR.Glutamic.acid | -0.0182 |
| UR.Tryptophan | -0.0183 |
| UR.2.3.phosphoglycerate | -0.0183 |
| UR.Methionine | -0.0189 |
| PL.CoA | -0.0189 |
| PL.Phenylalanine | -0.0197 |
| UR.ADP | -0.0212 |
| PL.Histidine | -0.0220 |
| UR.Valine | -0.0227 |
| PL.IMP | -0.0235 |
| PL.N.acetylglucosamine.1.phosphate | -0.0246 |
| UR.Citrulline | -0.0259 |
| UR.Nacetylneuraminate | -0.0266 |
| PL.Malate | -0.0298 |
| UR.NADH | -0.0299 |
| UR.FBP | -0.0299 |
| UR.GDP | -0.0301 |
| UR.UDP.D.glucuronate | -0.0301 |
| UR.CMP.N.acetylneuraminate | -0.0349 |
| PL.GAP.DHAP | -0.0382 |

| | |
|---|---|
| UR.Hexose.phosphate_3 | -0.0392 |
| UR.dCMP | -0.0394 |
| UR.Hexose.phosphate_1 | -0.0396 |
| PL.Succinate | -0.0443 |
| PL.Glycerol_3.phosphate | -0.0474 |
| UR.Bisphosphoglycerate | -0.0488 |
| PL.Citrate.isocitrate | -0.0608 |
| UR.Xanthosine | -0.0655 |

**Supplementary Table S7. MRM transitions for target metabolites.**

| | Retention time (min) | Cone voltage (V) | Precursor m/z | Collision energy (V) | Product m/z |
|---|---|---|---|---|---|
| **TQ-S:** | | | | | |
| 2+3-phosphoglycerate | 6.56 | 22 | 184.8 | 16 | 96.66 |
| 2-hydroxyglutarate | 6.29 | 35 | 146.94 | 15 | 84.814 |
| Acetyl-CoA | 8.29 | 40 | 808 | 37 | 408.01 |
| ADP | 7.14 | 34 | 426.12 | 21 | 158.62 |
| ADP-ribose | 6.92 | 50 | 558.1 | 21 | 345.78 |
| Allantoate_and_Carbamoyl_aspartate | 5.73 | 28 | 175 | 12 | 132 |
| AMP | 4.2 | 40 | 346.11 | 29 | 78.6 |
| ATP | 7.9 | 16 | 505.87 | 28 | 158.64 |
| Bisphosphoglycerate | 7.89 | 16 | 265 | 13 | 166.73 |
| CDP | 6.69 | 28 | 401.83 | 22 | 158.65 |
| Citrate/Isocitrate | 7.96 | 22 | 191 | 13 | 110.6 |
| CMP | 2.37 | 34 | 322.1 | 29 | 78.68 |
| CMP-N-acetylneuraminate | 6.37 | 40 | 613 | 20 | 322 |
| CoA | 8.18 | 40 | 766 | 40 | 408 |
| CTP | 7.78 | 10 | 481.8 | 22 | 158.67 |
| dCMP | 2.88 | 50 | 305.89 | 22 | 78.57 |
| FAD | 7.82 | 50 | 784.2 | 29 | 436.92 |
| FBP | 6.86 | 28 | 339 | 28 | 97 |
| Flavin_mononucleotide | 7.47 | 22 | 455 | 19 | 213 |
| Fumarate | 5.62 | 22 | 115 | 5 | 70.9 |
| GAP+DHAP | 2.56 | 16 | 169.05 | 13 | 97 |
| GDP | 6.9 | 28 | 441.8 | 16 | 343.78 |
| GDP-fucose | 6.8 | 40 | 587.937 | 25 | 441.728 |
| GDP-mannose | 6.54 | 10 | 603.908 | 30 | 423.691 |
| Glutathione_disulfide_oxidized | 6.04 | 46 | 611 | 21 | 306.01 |
| Glutathione_reduced | 2.15 | 40 | 305.89 | 16 | 142.72 |
| Glycerol_3-phosphate | 1.65 | 46 | 170.79 | 16 | 78.67 |
| GMP | 3.49 | 34 | 362.1 | 21 | 78.65 |
| Hexose-phosphate | 1.51 | 10 | 258.83 | 16 | 96.63 |
| IMP | 3.48 | 34 | 347.2 | 21 | 78.68 |
| Ketoglutarate | 6.27 | 22 | 145 | 5 | 100.77 |
| Malate | 5.61 | 16 | 132.77 | 16 | 70.69 |
| N-acetylglucosamine-1-phosphate | 1.77 | 22 | 299.9 | 22 | 78.78 |
| Nacetylneuraminate | 1.25 | 40 | 308 | 15 | 87 |
| NAD | 3.19 | 22 | 662.1 | 22 | 540.05 |
| NADH | 7.16 | 46 | 664.18 | 28 | 407.81 |
| NADP | 6.91 | 22 | 742 | 13 | 619.6 |
| NADPH | 7.91 | 34 | 744.07 | 34 | 407.83 |

| | | | | | |
|---|---|---|---|---|---|
| Phosphoenolpyruvate | 6.96 | 16 | 167 | 13 | 78.77 |
| Phosphogluconic_acid | 6.44 | 22 | 274.84 | 16 | 96.65 |
| Phosphoserine | 2.23 | 40 | 183.82 | 10 | 96.68 |
| Ribose-5-phosphate | 1.56 | 28 | 229 | 21 | 96.64 |
| Ribulose-5-phosphate | 1.92 | 28 | 229 | 21 | 96.64 |
| Succinate | 4.79 | 28 | 116.76 | 10 | 72.71 |
| UDP | 6.86 | 10 | 402.76 | 22 | 158.43 |
| UDP-D-glucose | 6.53 | 22 | 565 | 22 | 323 |
| UDP-D-glucuronate | 7.8 | 22 | 579 | 22 | 403 |
| UDP-N-acetylglucosamine | 6.57 | 30 | 606 | 25 | 385 |
| UMP | 3.19 | 46 | 322.9 | 28 | 78.55 |
| Uracil | 1.69 | 40 | 110.76 | 10 | 41.7 |
| UTP | 7.83 | 28 | 482.83 | 34 | 158.65 |
| Xanthosine | 1.55 | 28 | 283 | 22 | 151 |
| xylulose-5-phosphate | 2.27 | 28 | 229 | 21 | 96.64 |
| | | | | | |
| **Quattro Micro:** | | | | | |
| Alanine | 3.87 | 17 | 90.1 | 8 | 44 |
| Arginine | 7.5 | 24 | 175.1 | 18 | 70 |
| Asparagine | 2.04 | 19 | 133.1 | 14 | 74 |
| Aspartic acid | 1.68 | 19 | 134.1 | 10 | 74 |
| Citrulline | 4.83 | 16 | 176 | 28 | 70 |
| Glutamic acid | 2.37 | 19 | 148.1 | 14 | 84 |
| Glutamine | 2.47 | 16 | 147.1 | 14 | 84 |
| Glycine | 2.36 | 17 | 76 | 8 | 30 |
| Histidine | 7.39 | 20 | 156.1 | 12 | 110 |
| Isoleucine | 6.59 | 19 | 132.1 | 9 | 86 |
| Leucine | 6.59 | 19 | 132.1 | 9 | 86 |
| Lysine | 7.38 | 19 | 147.1 | 14 | 84 |
| Methionine | 5.96 | 19 | 150.1 | 9 | 104 |
| Ornithine | 7.33 | 16 | 133 | 16 | 70 |
| Phenylalanine | 6.66 | 20 | 166.1 | 10 | 120 |
| Proline | 5.35 | 21 | 116 | 10 | 70 |
| Serine | 2.02 | 19 | 106.1 | 10 | 60 |
| Threonine | 2.79 | 19 | 120.1 | 8 | 74 |
| Tryptophan | 6.72 | 19 | 205.1 | 14 | 146 |
| Tyrosine | 5.75 | 20 | 182.1 | 12 | 136.1 |
| Valine | 6.04 | 17 | 118.1 | 9 | 72 |
